# Metastatic Patterns and Adverse Histopathologic Features in Advanced Renal Cell Carcinoma: A Five-Year Single-Center Retrospective Pathology Study

**DOI:** 10.3390/medicina62050905

**Published:** 2026-05-07

**Authors:** Adelina Vidac, Alis Dema, Robert Barna, Aura Jurescu, Bianca Natarâș, Ioana Hurmuz, Diana Nicolcea, Vlad Dema

**Affiliations:** 1 ANAPATMOL Research Center, “Victor Babeș” University of Medicine and Pharmacy, 300041 Timișoara, Romania; adelina.slovac@umft.ro (A.V.); robert.barna@umft.ro (R.B.); jurescu.aura@umft.ro (A.J.); bianca.nataras@umft.ro (B.N.); ioana.hurmuz@umft.ro (I.H.); diana.nicolcea@umft.ro (D.N.); 2 Department of Pathology, “Pius Brînzeu” Emergency County Hospital, 300723 Timișoara, Romania; 3 Department of Urology, “Victor Babeș” University of Medicine and Pharmacy, 300041 Timișoara, Romania; vlad.dema@umft.ro

**Keywords:** renal cell carcinoma, clear cell renal cell carcinoma, metastatic renal cell carcinoma, metastatic patterns, pathology, retrospective study

## Abstract

*Background and Objectives:* Renal cell carcinoma (RCC) exhibits heterogeneous and sometimes unpredictable metastatic behavior, involving both common and uncommon anatomic sites. Institutional analyses of histopathologically confirmed metastatic RCC may improve diagnostic recognition and clinical awareness. This study aimed to characterize the metastatic distribution and histopathologic features of RCC diagnosed in a single tertiary center over a five-year period. *Materials and Methods:* A retrospective review of the pathology database of the Department of Pathology, “Pius Brînzeu” Emergency County Hospital, Timișoara, was performed to identify all histologically confirmed cases of metastatic RCC diagnosed between January 2020 and December 2024. Case identification was based on pathology reports of metastatic lesions. In a subset of cases, corresponding pathology reports of the primary renal tumor were available and reviewed. Histopathological data collected included WHO/ISUP grade, tumor necrosis, sarcomatoid and/or rhabdoid differentiation, vascular invasion, surgical margin status, tumor size, and pathological T stage (pT). Exploratory analyses were performed to assess associations between metastatic site and selected histopathological features. *Results*: Thirty-two cases of metastatic RCC were identified, all demonstrating clear cell morphology. The mean patient age was 62.9 years, with a marked male predominance. Among cases with available primary tumor data, high WHO/ISUP grade and adverse histopathologic features were frequently observed. The most common metastatic sites in our institution were the brain and bone, followed by the adrenal gland, lymph nodes, and liver. Less frequent metastatic involvement included the pancreas, testis, vagina, skin, and peritoneum. Exploratory analyses did not demonstrate statistically significant associations between metastatic site and tumor grade, necrosis, or sarcomatoid/rhabdoid differentiation; however, descriptive trends were observed, including the association of brain metastases with high-grade tumors and the high prevalence of tumor necrosis across metastatic sites. *Conclusions*: This pathology-based retrospective series highlights the broad metastatic spectrum of RCC, including both typical and rare anatomic sites. The predominance of clear cell morphology and the frequent association with adverse histopathologic features support the link between aggressive tumor biology and metastatic disease. Although no statistically significant associations were identified, the observed patterns suggest potential relationships between metastatic distribution and tumor characteristics, warranting further investigation in larger studies.

## 1. Introduction

Renal cell carcinoma (RCC) is the most common malignant tumor of the kidney in adults and accounts for approximately 2–3% of all cancers worldwide [1,2]. Its global incidence continues to rise, with more than 430,000 new cases and over 180,000 deaths reported annually, with higher rates observed in men and in highly developed regions [1,3]. Clear cell renal cell carcinoma (ccRCC) represents the predominant histologic subtype, comprising approximately 70–80% of all RCCs [2,4]. Despite advances in imaging, surgical management, targeted therapies, and immunotherapy, a substantial proportion of patients either present with metastatic disease at diagnosis or develop distant metastases during follow-up [5]. Metastatic RCC remains associated with an unfavorable prognosis, with reported five-year survival rates below 15% [6,7].

A hallmark of RCC is its heterogeneous and sometimes unpredictable metastatic behavior. The lungs, bone, lymph nodes, liver, adrenal glands and brain are the most frequently involved sites [6]. However, metastases may also occur in less typical locations, including the skin, pancreas, testis, female genital tract or the peritoneum, posing significant diagnostic challenges [8,9,10]. The mechanisms underlying this variability are incompletely understood and may involve organ-specific vascular patterns, tumor microenvironmental adaptations, and intrinsic biological features of RCC, although available evidence remains heterogeneous and occasionally conflicting [8,11]. Large population-based registries emphasize the predominance of pulmonary metastases in RCC [6,12]. In contrast, smaller pathology-based cohorts frequently report alternative metastatic distributions, influenced by referral patterns and case selection [13].

Recent advances in molecular characterization have further highlighted the role of angiogenesis-related pathways (e.g., VHL/HIF axis), immune microenvironment interactions, and genomic alterations in shaping metastatic behavior and organ-specific tropism in RCC, although their integration with histopathologic findings remains incomplete [14,15].

Histopathologic examination plays a central role in confirming the renal origin of metastatic lesions, particularly when tumors arise in unusual anatomic locations or display atypical morphology. Nevertheless, many published studies rely primarily on imaging findings or registry-based data, which provide limited histologic detail. Pathology-based series, although generally smaller in scale, offer unique insights into tumor grade, necrosis, sarcomatoid or rhabdoid differentiation, vascular invasion, and the histologic features of metastatic lesions—parameters closely linked to tumor aggressiveness and clinical outcome [16,17].

In this context, the present study provides a five-year, single-center, pathology-based analysis of histologically confirmed metastatic RCC. Unlike large registry-based studies, our approach focuses exclusively on histologically sampled metastatic lesions, allowing correlation with tumor grade, necrosis, and other adverse histopathologic features of the primary tumor where available. By emphasizing both common and uncommon metastatic sites within a pathology-driven cohort, this study aims to complement existing epidemiologic data and improve diagnostic awareness of atypical metastatic presentations.

## 2. Materials and Methods

We conducted a retrospective, observational study based on pathology records retrieved from the digital archive of the Department of Pathology, “Pius Brînzeu” Emergency County Hospital, Timișoara, Romania. Cases were identified through a systematic search of the database using diagnostic keywords such as “renal cell carcinoma”, “metastasis”, and “metastatic renal cell carcinoma”. All consecutive patients with histologically confirmed metastatic RCC diagnosed between January 2020 and December 2024 were included. Cases from 2020 were retained to ensure continuity of the five-year study period, despite the potential impact of the COVID-19 pandemic on healthcare access and diagnostic activity. Inclusion criteria required histopathologic confirmation of metastatic lesions of RCC origin. Cases involving primary renal malignancies other than RCC, including urothelial carcinoma, were excluded.

Primary tumor characteristics were available only for cases in which the initial surgical specimen was processed in our institution. Due to the lack of a centralized national cancer database and limited inter-institutional data access, complete clinical and pathological information could not be retrieved for patients treated elsewhere.

All tissue specimens were fixed in 10% neutral buffered formalin, routinely processed, embedded in paraffin, and sectioned at 3–4 μm, followed by hematoxylin and eosin (H&E) staining according to standard institutional protocols at the time of initial diagnosis. Histopathologic data were extracted from the original pathology reports archived in the institutional database, and no systematic slide review was performed for the purposes of this study. All diagnoses were established in routine clinical practice by board-certified pathologists, in accordance with the WHO Classification of Urinary System and Male Genital Organ Tumours.

Demographic data, including patient age and sex, were recorded for all cases. In cases with available primary tumor pathology reports, additional histopathological parameters were documented, including histological subtype, WHO/ISUP grade, presence of tumor necrosis, sarcomatoid and/or rhabdoid differentiation, vascular invasion, surgical margin status, primary tumor size, and pathological tumor stage (pT).

Immunohistochemistry (IHC) was performed in selected cases with atypical morphology or when confirmation of renal origin was required. Specifically, IHC was applied in a limited number of diagnostically challenging cases, including three brain tumors and one vaginal lesion. The antibody panels included renal markers such as PAX8, CAIX, and CD10 to support renal origin, along with site-specific markers, including GFAP and S100 in brain lesions, and estrogen receptor (ER) and p16 in the vaginal lesion. IHC findings were used exclusively for diagnostic purposes, and no additional immunohistochemical analyses were performed specifically for this study.

Statistical analysis was primarily descriptive. Categorical variables were reported as absolute numbers and percentages, while continuous variables were summarized as means and ranges. Results are presented in tables. Associations between metastatic site (brain vs. non-brain) and selected clinicopathological parameters, including tumor grade and the presence of tumor necrosis, were explored using contingency tables and Fisher’s exact test, given the small sample size.

As a single-center, pathology-based study, the cohort reflects the spectrum of histologically sampled lesions within our institution and may be influenced by referral patterns and local clinical practices. In particular, the availability of certain surgical specialties, such as thoracic surgery, may influence the spectrum of histologically sampled lesions, potentially contributing to the underrepresentation of pulmonary metastases in our cohort.

## 3. Results

### 3.1. Patient Demographics

A total of 32 patients with histologically confirmed metastatic RCC were included. All metastatic lesions demonstrated clear cell morphology, and no non-clear cell RCC subtypes were identified. Patient age ranged from 43 to 83 years, with a mean age of 62.9 years. The cohort showed a marked male predominance, with 25 males (78.1%) and 7 females (21.9%) (Table 1).

### 3.2. Primary Tumor Characteristics

Pathological data regarding the primary renal tumor were available for 16 of the 32 cases (Table 2). All evaluable primary tumors demonstrated clear cell morphology. WHO/ISUP grading revealed grade 4 tumors in 10 cases (62.5%), grade 3 tumors in 5 cases (31.3%), and grade 2 tumors in 1 case (6.2%). Tumor necrosis was present in 11 cases (68.8%) and absent in 5 cases (31.2%). Sarcomatoid and/or rhabdoid differentiation was identified in 9 cases (56.3%) and was not observed in 7 cases (43.7%). Vascular invasion was reported in 13 cases (81.3%) and was absent in 3 cases (18.7%). Pathological tumor stage included 15 tumors classified as pT3a (93.8%) and one pT1a tumor (6.2%) resected by partial nephrectomy. Surgical margin status was positive in 4 cases (25.0%) and negative in 12 cases (75.0%). Primary tumor size data were available for all evaluable primary tumors, with a mean diameter of 9.87 cm.

### 3.3. Metastatic Distribution

The most frequent metastatic sites were the brain (10 cases, 31.3%) and bone (9 cases, 28.1%). Brain metastases involved the bulbar region in 1 case (10.0% of brain metastases), the frontal lobe in 3 cases (30.0%), the frontoparietal lobes in 2 cases (20.0%), while the specific intracranial location was unknown in 4 cases (40.0%). Bone metastases affected the left iliac crest in 1 case (11.1% of bone metastases), the cervical spine in 3 cases (33.3%), the thoracic spine in 3 cases (33.3%), the sacral spine in 1 case (11.1%), and the left frontal bone with orbital involvement in 1 case (11.1%). Additional metastatic sites included the adrenal gland (4 cases, 12.5%), lymph nodes (2 cases, 6.3%; one cervical and one unknown), liver (1 case, 3.1%), pancreas (1 case, 3.1%), testis (1 case, 3.1%), skin (1 case, 3.1%), vagina (1 case, 3.1%), peritoneum (1 case, 3.1%) and omentum (1 case, 3.1%) (Table 3).

### 3.4. Metastatic Site and Histopathological Correlates

Associations between metastatic site (brain vs. non-brain) and selected histopathological features were explored in the subset of cases with available data.

No statistically significant association was observed between metastatic site and tumor grade (Fisher’s exact test, *p* = 1.0). However, all brain metastases in this subset were associated with high-grade tumors. Similarly, no statistically significant association was identified between metastatic site and the presence of tumor necrosis (Fisher’s exact test, *p* = 1.0). Tumor necrosis was observed in a high proportion of cases in both groups, being present in 66.7% of brain metastases and 80% of non-brain metastases.

Analysis of sarcomatoid and/or rhabdoid differentiation also did not reveal a statistically significant association with metastatic site (Fisher’s exact test, *p* = 0.30). However, these features were more frequently observed in non-brain metastases (70%) compared to brain metastases (33.3%).

## 4. Discussion

This five-year pathology-based retrospective study analyzed 32 patients with histopathologically confirmed metastatic RCC, all of whom demonstrated clear cell morphology. In cases with available primary tumor pathology, the corresponding renal tumors also exhibited clear cell histology, consistent with the known predominance of this subtype in advanced RCC [18]. The demographic profile of the cohort, with a mean age of 62.9 years and a marked male predominance, is concordant with large epidemiological studies reporting peak incidence in the sixth to seventh decades of life and a male-to-female ratio of approximately 2:1 [19,20].

In the present series, the most frequent metastatic sites were the brain and bone, reflecting the distribution of pathologically sampled cases in our institution, which differs from the classic metastatic pattern of RCC, in which pulmonary involvement predominates and is reported in up to 45–55% of cases [6,21]. This discrepancy is largely attributable to the pathology-based design of the study and to institutional factors, most notably the absence of a thoracic surgery unit. As a result, pulmonary metastases—frequently diagnosed radiologically and often managed without surgical resection—are rarely available for histopathologic evaluation in our setting. Consequently, lung metastases are underrepresented in this cohort.

Brain metastases were identified in 10 patients (31.3%), most frequently involving the frontal and frontoparietal regions, with additional cases affecting the bulbar region or reported at unspecified intracranial locations. This proportion exceeds the 8–10% incidence typically reported in population-based cohorts and may reflect improved neuroimaging surveillance and increased recognition of central nervous system involvement in advanced disease [6,7,12]. Bone metastases were documented in 9 patients (28.1%), a frequency comparable to the 30–35% reported in large series [6,22]. The axial skeleton was predominantly involved, particularly the cervical and thoracic spine, with additional involvement of the sacral spine, iliac crest, and a single case of frontal bone metastasis with orbital extension. Adrenal metastases accounted for four cases (12.5%), consistent with previously reported rates ranging from approximately 8% to 14% [6,12]. Only one hepatic (3.1%) and two nodal (6.3%) metastases were identified, proportions that are lower than those usually reported in large series, a difference that should be interpreted in the context of the limited size and pathology-based design of this cohort [6,11].

Several rare metastatic sites were identified in our cohort, including the testis, vagina, skin, peritoneum, omentum, and pancreas, underscoring the broad and sometimes atypical metastatic potential of RCC. Although individually uncommon, these sites are recognized in the literature as manifestations of advanced disease and may present diagnostic challenges, particularly when metastatic clear cell morphology mimics site-specific primary tumors. Notably, the identification of vaginal and testicular metastases in our series—both exceptionally rare entities—highlights the importance of considering RCC in the differential diagnosis of unusual lesions [9,10,23,24,25]. The presence of pancreatic, cutaneous, and peritoneal metastases in our cohort further supports the well-documented capacity of RCC for organ-selective and atypical metastatic spread [26,27,28,29,30,31,32].

The histopathologic features observed in evaluable primary tumors suggest a predominantly aggressive disease phenotype. Among the 16 cases with available primary tumor data, high WHO/ISUP grade was common, with grade 4 tumors accounting for more than half of the cases. Tumor necrosis, sarcomatoid and/or rhabdoid differentiation, and vascular invasion were frequently identified—features that have been consistently associated with increased metastatic potential and adverse clinical outcomes in prior studies [16,17]. The predominance of pT3a tumors (93.8%) and the large mean primary tumor size (9.87 cm) further support the advanced nature of disease in this cohort, in line with published evidence linking tumor size and local extension to metastatic risk [33,34]. However, survival and treatment-related data were not available in the present study, precluding direct assessment of prognostic outcomes and limiting the interpretation of these findings within our cohort.

Exploratory analyses were performed to assess potential associations between metastatic site and selected histopathological features. Although no statistically significant associations were identified between metastatic site (brain vs. non-brain) and tumor grade, tumor necrosis, or sarcomatoid/rhabdoid differentiation, likely due to the limited sample size, several descriptive trends were observed. Notably, all brain metastases in the analyzed subset were associated with high-grade tumors, supporting a potential link with more aggressive tumor biology. In contrast, sarcomatoid and/or rhabdoid differentiation was more frequently observed in non-brain metastases, suggesting possible heterogeneity in the pathways of tumor progression and dissemination. Tumor necrosis was highly prevalent across both groups, further supporting its role as a marker of aggressive disease in metastatic RCC.

Patterns of metastatic dissemination in RCC have been shown to correlate with prognosis, with brain and bone involvement generally associated with worse outcomes compared with isolated pulmonary or nodal metastases [6,12,35]. The predominance of these sites in the present cohort therefore suggests a biologically aggressive subset of disease. Although survival data and treatment information were not uniformly available, the frequent association with high-grade histology and adverse pathologic features supports the concept that both metastatic pattern and tumor biology contribute to risk stratification in metastatic RCC.

This study has several limitations, including its retrospective design, single-center setting, and relatively small sample size, which may limit generalizability. A key limitation is the pathology-based design and the absence of a thoracic surgery department at our institution, which resulted in the underrepresentation of lung metastasis in this series. In addition, the incomplete availability of primary tumor characteristics—due to limited access to external medical records in the absence of a centralized national cancer registry—may introduce selection bias. Nevertheless, this study provides valuable insight into the metastatic spectrum of RCC through tissue-verified diagnoses and highlights both common and rare metastatic presentations encountered in routine diagnostic practice.

## 5. Conclusions

This five-year, pathology-based series highlights the heterogeneous metastatic behavior of RCC, with a predominance of brain and bone involvement among histologically sampled metastases in our institution, and occasional dissemination to rare anatomic sites, including the vagina, testis, skin, and pancreas. The frequent association with adverse histopathologic features underscores the importance of comprehensive pathologic evaluation and heightened awareness of atypical metastatic presentations in routine diagnostic practice. Exploratory analyses further suggest that metastatic patterns may be associated with underlying tumor biology, although these findings require validation in larger cohorts.

## Figures and Tables

**Table 1:** Patient demographics.

Sex	*n* (%)
Male	25 (78.1%)
Female	7 (21.9%)

**Table 2:** Histopathological characteristics of available primary tumors (*n* = 16).

Parameter	Category	*n* (%)
Histological subtype	Clear cell	16 (100%)
WHO/ISUP grade	2	1 (6.3%)
	3	5 (31.3%)
	4	10 (62.5%)
Tumor necrosis	Present	11 (68.8%)
	Absent	5 (31.2%)
Sarcomatoid/rhabdoid differentiation	Present	9 (56.3%)
	Absent	7 (43.7%)
Vascular invasion	Present	13 (81.3%)
	Absent	3 (18.7%)
Pathologic T stage (pT)	pT1a	1 (6.3%)
	pT3a	15 (93.7%)
Surgical margin status	Negative	12 (75.0%)
	Positive	4 (25.0%)
Primary tumor size (cm)	Mean	9.87

**Table 3:** Anatomic distribution of metastatic sites.

Metastatic Site	*n* (%)	Subsite
Brain	10 (31.3%)	Bulbar (1), frontal (3), frontoparietal (2), unknown (4)
Bone	9 (28.1%)	Iliac crest (1), cervical spine (3), thoracic spine (3), sacral spine (1), frontal bone/orbit (1)
Adrenal gland	4 (12.5%)	-
Lymph nodes	2 (6.3%)	-
Liver	1 (3.1%)	-
Other rare sites	6 (18.7%)	Pancreas (1), testis (1), skin (1), vagina (1), peritoneum (1), omentum (1)

## Data Availability

Data are available upon reasonable request.

## Abbreviations

The following abbreviations are used in this manuscript:

RCC	Renal cell carcinoma
ccRCC	Clear cell renal cell carcinoma
IHC	Immunohistochemistry
H&E	Hematoxylin and eosin
WHO	World Health Organization
ISUP	International Society of Urological Pathology
